# Ant Diversity and Community Composition in Alpine Tree Line Ecotones

**DOI:** 10.3390/insects12030219

**Published:** 2021-03-04

**Authors:** Elia Guariento, Konrad Fiedler

**Affiliations:** 1 Department of Botany & Biodiversity Research, University of Vienna, Rennweg 14, 1030 Vienna, Austria; elia.guariento@eurac.edu; 2 Institute for Alpine Environment, Eurac Research, Viale Druso 1, 39100 Bolzano, Italy; 3 Department of Ecology, University of Innsbruck, Sternwartestrasse 15, 6020 Innsbruck, Austria

**Keywords:** ant ecology, biodiversity, alpine ecology, functional diversity, functional traits, elevation gradients

## Abstract

**Simple Summary:**

Alpine grasslands are among the few terrestrial habitats not obviously dominated by ants. Yet few studies have addressed how ant communities change along tree-line ecotones on mountains. We combined five survey methods to assess ant assemblages along the tree line at five mountains in the south-central Alps of Italy. Ant species richness peaked directly at the tree line, but this was not due to a mixing of forest with grassland species. In subalpine forest and at the tree line, ant assemblages were dominated by mound-building red wood ants. Community composition and functional species traits indicated competition as a potential effect of community assembly in subalpine forest. Further, habitat features such as elevation, dwarf shrub cover, and the extent of a soil humus layer shaped species composition of ant communities around the tree line.

**Abstract:**

Ants are crucial for the functioning of many terrestrial ecosystems, but detailed knowledge of their ecological role is often lacking. This is true for high mountains where a steep environmental gradient exists from mountainous forest, densely populated by ants, to grassland habitats above the tree line, harboring a sparse ant community. We assessed ant communities in and around the tree line ecotone on five slopes in the southern-central Alps, focusing on their species diversity, community composition, and functional dimensions. Species richness and functional diversity were highest directly at the ecotone. Ant community composition was shaped by elevation and shrub cover. Further, the abundance of the dominant mound-building red wood ants (*Formica* s. str.) influenced the community composition of the subordinate species. We conclude that over the tree line ecotone a shift in predominance from biotic limitations in the forest to abiotic filters in the alpine environment takes place.

## 1. Introduction

Ants have a predominantly thermophile geographic distribution and their centers of origin as well as of current diversity are located in the tropics [1,2]. Distributional limits of some ants, however, reach far into colder biomes such as boreal and subarctic coniferous forests, the tundra [3,4], and also into alpine grassland above the tree line [5,6]. Elevational gradients are often chosen to study the drivers behind distributional borders and diversity patterns [7,8], because here important life shaping factors change over small distances. Those factors are essentially linked to the strong decrease of ambient temperatures at high elevations, that influences the duration of the growing season and net primary production [9]. Ant diversity decreases at high elevations, with some studies describing a linear decrease [10,11] while others found a hump shaped relation with diversity peaking at intermediate elevation [12]. Reymond et al. [5] also observed a reduction of functional diversity with increasing elevation in the European Alps, and a later study [13] found a similar pattern for ant phylogenetic diversity along three elevation gradients in Europe and North America.

Studies identified temperature as main factor limiting ant diversity on elevational gradients [14,15]. However, a very exhaustive review suggests that a complex interplay of several drivers limits ant diversity at high elevations [12] with a massively reduced diversity observed at all high elevation sites around the world. Nevertheless, the concordant decrease of all diversity measures with elevation suggests that a set of strong habitat filters selects for few, and often related, ant species [13]. These species are expected to share predictable trait combinations that enable them to survive in these harsh environments [5]. Using a functional trait and diversity approach is thus a promising way to detect potential constrains acting on a community [5,16].

The alpine grassland belt represents the elevational distribution limit for most ant species in Europe [17,18]. Only few species (in Austria, for example, less than 20 species [19]) occur regularly above the tree line, leaving alpine (or arctic) habitats among the few terrestrial ecosystems where ant prevalence is limited. Alpine environments will be most severely affected by climate and land-use change in the near future [20,21]. Since temperature is the main limiting factor for the position of the tree line ecotone [22] and for the occurrence of ants at high elevations, elevational distributions of trees as well as ants will probably change in the next few decades [23]. This underlines the importance of investigating the present status of the ant community around the tree line ecotone.

Studies dealing with ant fauna along forest-grassland intersections are sparse. In the tropics, a high species richness at ecotones was found [24], but this pattern is not universal [25]. In temperate regions, high species richness was found on a grassland-to-shrub edge [26] and behaviorally dominant ants increase in presence on agricultural field margins all over Central Europe [27]. 

Regarding the ant community composition on the elevational tree line in temperate regions, very little information is available. However, comparing studies from truly alpine settings with those conducted in montane forest below [5,6,28,29,30] leads to some expectations. Abiotic environmental filtering is expected to prevail in the alpine grassland, where a harsher environment is selecting for fewer but well adapted species [13]. Inside the subalpine forest a stronger competition among ants can be expected [29] to influence the community composition, since mound-building wood ants (*Formica* s. str. Linnaeus, 1758) are present there in abundance [31,32]. Wood ants are the dominant ant group in coniferous forests in boreal or mountainous environments across Europe [33]. They are associated with the presence of trophobiotic partners (in this case mostly *Cinara* Curtis, 1835 aphids that feed on conifers and provide access to plant phloem for ants that in return tend and protect the aphids [34]), enabling them to maintain great colony sizes and dense populations by providing access to plant derived carbohydrates [35]. In a previous study over the same alpine tree line ecotones, we showed that the availability of trophobionts and the abundance of wood ants decreased from the forest to the alpine habitat above the tree line [31].

The two forces that shape ant community composition over this ecotone are therefore expected to shift from a prevalence of biotic competition among ants (niche partitioning effect) in the forest to environmental abiotic limitation above the tree line (niche filtering effect). We expect the shift in ant community ecology to mirror this change of limiting factors in all the investigated dimensions. Specifically, we expect (1) to find an increase in species richness at the ecotone between the forest below and the alpine environment above the tree line as a result of faunal mixing between the habitat types. Further, we expect (2) the ant community composition to reflect the habitat conditions and to change accordingly over the ecotone. We also expect (3) that shifts in the prevalence of single functional traits describes more in detail the constraints inherent to the ant community along this environmental gradient. Finally (4), functional richness is expected to peak at the ecotone in concordance to the overall picture of a high ant diversity directly at the ecotone sites and a higher functional divergence (i.e., broader occupied niche space) is expected to occur in the forest where multiple co-occurring species try to avoid competition, while low values are expected where strong habitat filtering effects force species to converge in trait space.

## 2. Materials and Methods 

### 2.1. Study Sites

**Figure 1:**
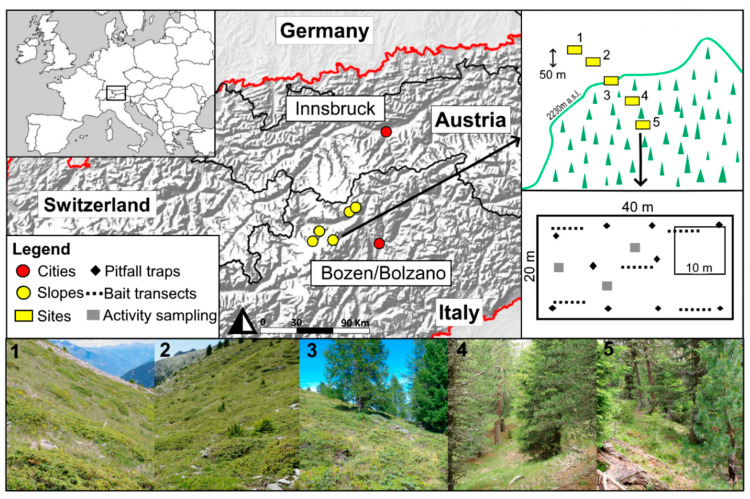
Map of the study sites depicting the five slopes. The inset on top right is an example of the sites’ position within one slope. The numbering of the single sites matches the numbers of the pictures giving an overview about the single sites and the habitats covered by one slope. Numbers 1 and 2 depict the alpine habitat characterized by low vegetation interspersed by dwarf shrubs. Number 3 is an example of the ecotone site. Numbers 4 and 5 depict the forest sites with a denser tree cover. An additional inset (middle right) shows schematically the distribution of all samples taken with five survey methods within each site.

Sampling was conducted on five slopes of the southern part of the Central siliceous Alps, in South Tyrol (Italy). To facilitate comparisons among sites, only south facing slopes within natural reserves were chosen (Texelgruppe, two slopes; Stilfserjoch, three slopes). Each selected slope revealed a gradient of near-natural vegetation from subalpine coniferous woodland to open alpine grassland interspersed with shrubs, without major changes in exposition and steepness (Figure 1). The tree line itself was defined by a sharp edge between forest and subalpine environment, as a result of management that occurred in the past and still persists in the form of extensive summer pasturing by cattle [36].

We defined five sampling sites (of 800 m^2^ area each) on each slope. They were placed along an elevational gradient, with a distance of at least 50 elevation meters between adjacent sampling sites and with the tree line serving as the center at each slope. Thus, two sampling sites per slope were located inside the subalpine forest, one was situated exactly on the tree line ecotone, and two were in the alpine grassland. Each slope covered roughly 200 m of elevation, and overall sites spanned 470 m from 1935 m (at the lowest site) to 2405 m (at the highest site). Ant sampling took place in July and August 2016 and 2017. Within each site in a 10 × 10 m square positioned to cover average site characteristic (e.g., avoiding anomalies such as eroded patches, fallen trees, tree gaps on the forest), we estimated the cover (in %) of the main plant strata (e.g., trees, dwarf shrubs, herb vegetation), of open soil surface, rocks, and dead wood. Moreover, a list of all vascular plant species for each site was compiled and their Landolt indicator values of light and soil humus were extracted from [37] (Table S1; for further habitat descriptors and the plant species lists of each site see [38]).

### 2.2. Sampling

To assess ant communities, the use of several sampling methods in parallel can help to gain a more complete and representative coverage of species [39,40]. Following this rationale, we implemented five standard sampling methods for ants simultaneously, namely, baiting, pitfall traps, quadrat sampling, colony sampling, and hand sampling of foraging worker ants. Each method has its advantages and disadvantages (as discussed by [41]). We used the incidence of records of a given ant species at each site with each method (yielding a possible maximum score of five for every species per site) as a ‘pseudo-abundance’ score. This score reflects the ecological prevalence of each ant species among the sites, but levels out imbalances in observed worker ant numbers that may happen due to mass recruitment, ant trail vicinity, or under very favorable weather conditions.

Sampling occurred during the time of peak ant activity in alpine habitats, between 9 am and 4 pm. Baiting occurred in transects offering six different liquid resources (sucrose, the amino acid glutamine, sucrose-glutamine mixture, salt, plant oil, and pure water, following a well-established protocol [42,43]) and exposed for at least 3 h. Resources were offered in 50 mL plastic centrifuge tubes placed on the ground with a cotton ball soaked in 10 to 20 mL of the liquid bait. Seven transect lines of baits (each transect with one bait per resource type) were placed with at least 10 m distance from each other on each site. The retrieval of some baits occurred only after 6 pm because of the remoteness of the locations. Ten pitfall traps (plastic tubes with of 3 cm diameter opening and 7 cm deep) were placed on each site, spaced by ca. 10 m from another and exposed for three consecutive days. The traps were partially filled with a baiting liquid composed of 1:1 mixture of rum and honey and few drops of a detergent to reduce surface tension (demonstrated to be effective in [44]). Activity density of foraging workers outside their nests was quantified with a fixed frame (50 cm × 50 cm) set on the ground. All ants within or entering the frame for 10 min were counted and identified. Three replications of this method per site were used. Colony sampling occurred inside a fixed area (10 m × 10 m) wherein all colonies of smaller ant species were counted during an exhaustive search. The fixed area was placed within each site covering an average configuration of the whole site (e.g., on the tree line containing at least one medium tree and also open areas). For bigger ant species (such as mound-building wood ants), all nest mounds were counted inside the whole sampling area (800 m^2^). Finally, hand sampling of worker ants encountered on the soil surface or on the vegetation during 20 min of intense search was intended to complement the species list of each site. The full sampling design was done in 2016, while in 2017 only the bating method was replicated, and the data were pooled with the first sampling year.

Subsequently, voucher specimens collected were identified to species level using the keys in [45] and [46], with the help of a stereo-microscope (90-fold magnification) connected to a computer (Table S2).

### 2.3. Statistics

Observed species richness, functional diversity and community weighted means of selected species traits were analyzed by linear mixed-effect models (LMM) in the *R* environment [47] using the *lme4* package [48]. Slope identity was modelled as random factor to account for possible spatial autocorrelation. Habitat type (with the 3 categories alpine/tree line/forest) was set as fixed factor. To test the effect between two habitats, we ran a reduced LMM excluding one habitat. Results were visualized using *ggplot2* [49]. Sampling coverage and species accumulation curves per habitat type were computed to visualize the sampling effectiveness and comparing species pools among habitats. We used the R package *iNEXT* [50] with the species incidences obtained with all five sampling methods on each site serving as replicate units of observation (i.e., yielding 25 observation units on the tree line ecotones and 50 units in the alpine and forest habitats, respectively).

The differences among the communities in regard to the habitat categories were investigated using a PERMANOVA (*adonis* function in package *vegan* [51]) based on a Bray-Curtis distance matrix with 999 permutations.

Community composition and its potential drivers were further analyzed with a Canonical Analysis of Principal coordinates (CAP), using the function *capscale()* in the package *vegan* [51]. For these analyses, we used the pseudo-abundance score described before. First, the whole ant community was investigated to identify important habitat factors, then the same procedure was applied upon the community without the wood ants. For this additional analysis, we were particularly interested in how the community of subordinate ant species is shaped by the prevalence of the dominant mound-building red wood ants. We therefore used the pseudo-abundance of mound-building wood ants, *F. lugubris* Zetterstedt, 1838 and *F. aquilonia* Yarrow, 1955, as explanatory variable for the subdominant fraction of the ant community. Bray-Curtis distances were used as basis for these analyses of community composition. An additional dummy species with abundance 1 at every site was added to the community of subordinate species to improve the stability of the ordination [52], given the partially sparse ant species lists per site. All predictor variables were *z*-transformed prior to inclusion in statistical models to avoid scaling issues. Four explanatory variables (wood ant pseudo-abundance, average humus indicator value of the local vegetation, mean plot elevation, and dwarf shrub cover of a 10 × 10 m subplot) were initially chosen, based on their putative ecological relevance. After constructing the full model, we simplified this statistical model using a combination of criteria. First, the Akaike information criterion corrected for small sample sizes (AICc) was used to determine a set of the best models (only a difference of AICc values > 2 was considered as improving model fit). Then, Akaike weights [53] and R^2^ values (function *RsquareAdj()* in *vegan*; [54]) were used to finally decide which set of variables optimally explained the ant community composition data.

Seifert [18] collected a plethora of information about the ecology and distribution of European ant species. We used this information, supplemented by scores of the observed ant species’ position in competition hierarchies taken from [33], to construct a species × trait matrix. Altogether, information on 28 traits (subdivided into seven categories) describing eco-morphological characteristics like queen and worker size, nest characteristics, colony founding strategy, feeding habits, foraging behavior, and dominance aspects of all observed ant species were collected (Table S3). We chose a subset of traits to be visualized as community weighted means (CWM), because of their consistency with the argumentation that competition and resource limitation might change over the investigated ecotone. These included the contribution of the two most important nutrient sources (trophobiosis versus carnivorous lifestyle) to the nourishment of ant species, one morphological trait (CS: the arithmetic mean of maximum measurable head length and width as measure for head size), colony size (worker number in a mature colonies), and position of species in the dominance hierarchy (as assigned in [33]).

For the computation of community-weighted means and multivariate functional diversity indexes (according to [55,56]) the package *FD* [57] was used. Especially the measurement of functional richness and functional divergence (presented also graphically in the results) were of major interest.

## 3. Results

### 3.1. Species Richness and Diversity

Approximately 23,500 individual ants were collected by all sampling methods, representing 13 different species (Table S2). The sampling methods yielding most species were pitfall traps and hand sampling (12 species each), the others followed closely with colony sampling yielding the lowest number of nine species. Most ant species (eight) occurred over the whole investigated gradient. Four species occurred only within the subalpine forest (*Camponotus herculeanus* (Linnaeus, 1758), *Myrmica rubra* (Linnaeus, 1758), *Formica aquilonia* and *Myrmica lobicornis* Nylander, 1846), while *Formica exsecta* Nylander, 1846 occurred only at and above the tree line.

**Figure 2:**
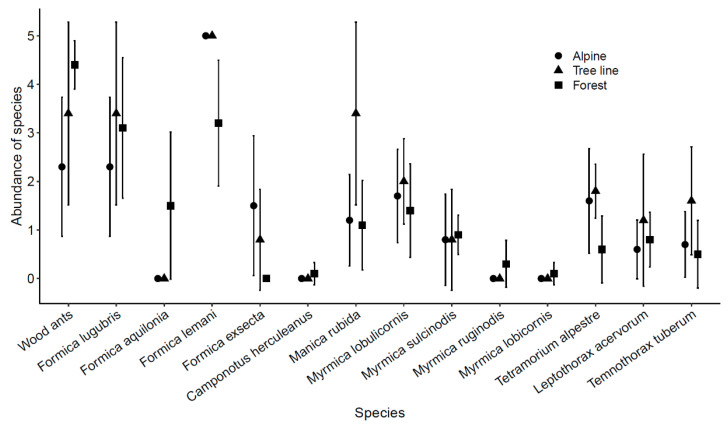
Mean pseudo-abundance of ant species with 95% confidence intervals split among the three habitat types. The maximum possible abundance score was five, only reached on average by *Formica lemani* in alpine grassland and on the tree line. The sum of pseudo-abundances of all mound-building wood ants together (comprising *F. lugubris* and *F. aquilonia*) was included to visualize the overall prevalence of these two ant species which otherwise tend to exclude each other locally.

The pseudo-abundance of ant species delivers a clear picture about the dominant ones and the species distribution over the investigated gradient (Figure 2). The subgenus of mound-building wood ants (*Formica* s. str.) dominated numerically in the forest (LMM: Alpine/Forest Chi^2^ = 9.105, *p* = 0.003) and similarly on the tree line (Tree Line/Forest Chi^2^ = 3.286, *p* = 0.070.), while *Formica lemani* Bondroit, 1917 was the most abundant species on the tree line and above (LMM: Alpine/Forest Chi^2^ = 8.730, *p* = 0.003; Tree Line/Forest Chi^2^ = 4.666, *p* = 0.031). Five of eight Myrmicinae species were more abundant at the tree line sites than elsewhere (sum Myrmicinae species abundance: LMM: Alpine/Tree line Chi^2^ = 6.628, *p* = 0.010; Tree Line/Forest Chi^2^ = 4.863, *p* = 0.027).

**Figure 3:**
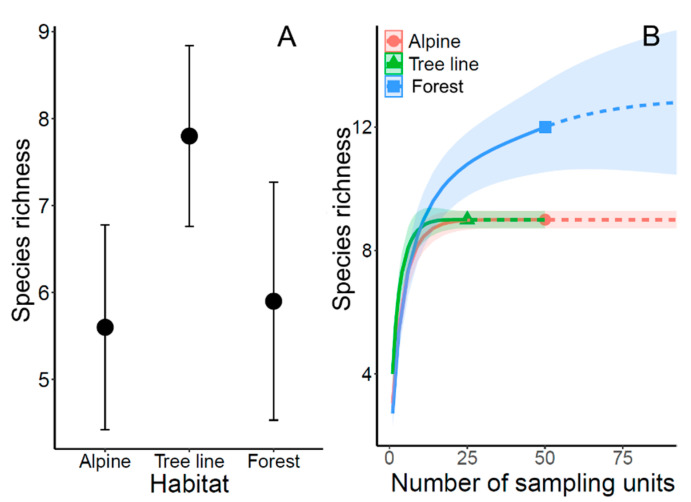
(**A**) Mean ant species richness among the three habitat types. Whiskers show the 95% confidence intervals. (**B**) Species accumulation curves as a function of sampling units, based on ant species incidences. Symbols: observed species richness. Dashed lines: extrapolated species richness. Shaded areas: 95% confidence limits of the curves. In subalpine forest, ant species richness was distinctly higher than on the tree line and in alpine grassland, and some further species might have been detected with more intense sampling in forest.

As expected, we observed higher species richness at the ecotone (LMM: Alpine/Tree line Chi^2^ = 7.53, *p* = 0.006; Tree Line/Forest Chi^2^ = 4.605, *p* = 0.032; Figure 3) than in the adjacent alpine or forest habitat. This result was not caused by faunal mixing from the two adjacent habitat types, but by a high incidence of several ant species at the ecotone, especially of the Myrmicinae subfamily (Figure 2). The expected faunal mixing would have resulted if species occurred predominantly in one habitat and the ecotone, but most species spread over the entire investigated gradients (Figure 2).

Species accumulation curves were computed to (1) determine how complete the sampling of ants was in the three habitat types and (2) how many species were to be expected in total. For both the tree line and the alpine grassland a very high, near complete coverage was obtained. In contrast, in the subalpine forest, some ant species might have still been missed by our sampling design (Figure 3).

### 3.2. Community Composition

Communities of ants differed significantly between forest and alpine habitats (PERMANOVA, 999 permutations: F_1,19_ = 4.29, *p* = 0.002). However, we recorded no significant difference between tree line and forest (PERMANOVA, 999 permutations: F_1,14_ = 2.797, *p* = 0.051), or between tree line and alpine communities (PERMANOVA, 999 permutations: F_1,14_ = 1.643, *p* = 0.166).

**Figure 4:**
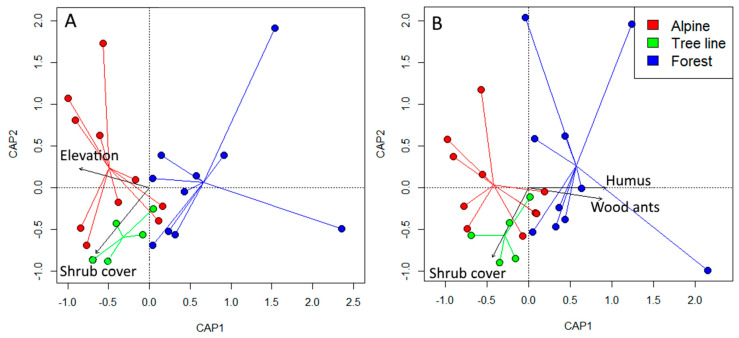
Ordination plots from the capscale routine. (**A**) The influence of the most important explanatory variables (viz. dwarf shrub cover and elevation) upon the full community of ants. (**B**) Ordination showing the species composition of subdominant ant assemblage and the most important explanatory variables (viz. wood ant abundance, dwarf shrub cover and soil humus indicator value of the vegetation). The mound-building dominant wood ant species were here excluded from the ant community matrix and their abundance score served as additional explanatory variable for the remaining subordinate fraction of the ant community. Ant communities from alpine grassland and forest sites cluster apart from another. Those from tree line sites cluster more with the alpine sites and display a reduced dispersion. Spider web centers represent the weighted centroids of community samples for each habitat type.

The best model fit for the complete ant community resulted with elevation and dwarf shrub cover as significant explanatory factors that influence community composition (Table 1; Figure 4). Nonetheless, similar model fits were detected also when including the average soil humus Landolt value derived from the local plant species lists.

The best model for the subordinate ant community (excluding the wood ants) revealed a similar outcome, with dwarf shrub cover and the average soil humus value as main explanatory factors. The abundance of wood ants, here used as additional explanatory factor, clearly influenced the subordinate ant community composition. Additionally, incorporating elevation of the sites in the model increased the fraction of explained variance, but at the cost of increasing AICc. Overall, species composition of non-dominant ant assemblages in subalpine forest was strongly related to high wood ant presence and high soil humus values, whereas tree-line ant communities were associated with high dwarf shrub cover (Table 1).

### 3.3. Functional Diversity and Traits

**Figure 5:**
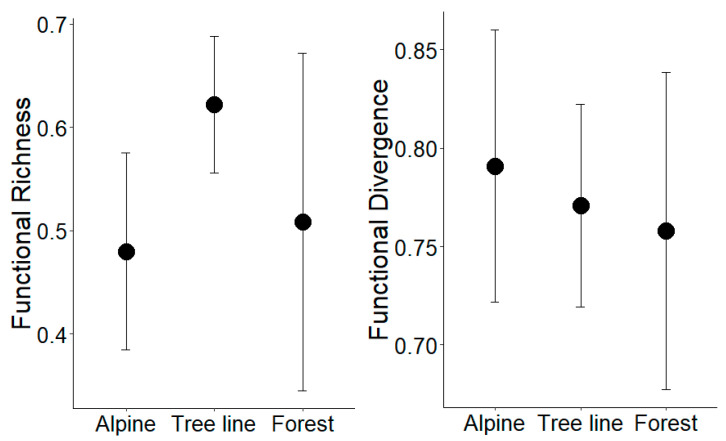
Means ± 95% confidence intervals of two functional diversity measures, functional richness and functional divergence, based on a matrix of 28 traits per species. A higher score on the tree line compared to the alpine sites was detected for functional richness only.

Functional richness scored significantly higher at the tree line habitats than in the alpine habitat (Figure 5; LMM: Alpine/Tree line Chi^2^ = 4.98, *p* = 0.026), but not in comparison to the forest (LMM: Forest/Tree line Chi^2^ = 1.73, *p* = 0.188), or between alpine and forest sites (LMM: Alpine/Forest: Chi^2^ = 1.68, *p* = 0.682). Functional evenness (FEve) and functional dispersion (FDis) revealed largely the same pattern as functional richness, but without a significant difference (data not shown). Functional divergence (FDiv) did not reveal, as expected, a higher score at the forest sites, compared to both alpine grassland and tree line sites (LMM: Forest/Alpine Chi^2^ = 0.56, *p* = 0.453; Forest/Tree line Chi^2^ = 0.07, *p* = 0.792; Figure 5).

**Figure 6:**
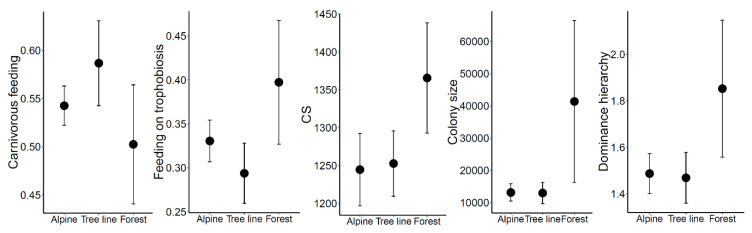
Plots of community weighted means and their 95% confidence intervals of some important functional traits of ant species. For all depicted traits, significant differences (*p* < 0.05) among habitats were detected using linear mixed models with habitat as fixed factor and slope modelled as random factor.

CWM analyses showed that forest communities engaged more intensely in trophobiosis than in the alpine setting (LMM: Alpine/Forest Chi^2^ = 5.81, *p* = 0.016), or at the tree line (LMM: Tree line/Forest Chi^2^ = 5.07, *p* = 0.024; Figure 6). We observed the opposite pattern for predation, but a significant difference was only detected between tree line and forest communities (LMM: Tree line/Forest Chi^2^ = 4.39, *p* = 0.036; Figure 6). Ant assemblages directly at the tree line displayed the strongest expression of carnivory and the lowest average role of trophobiosis. This pattern probably reflects the high presence of species of the Myrmicinae subfamily (many of which have more carnivorous feeding habits; Table S3). Subalpine forest ant communities consisted of larger species (LMM: Alpine/Forest Chi^2^ = 10.56, *p* = 0.001; Tree lien/Forest Chi^2^ = 5.40, *p* = 0.02; Figure 6) with far larger colony sizes than alpine communities (LMM: Alpine/Forest Chi^2^ = 6.17, *p* = 0.013; Figure 6) and attaining higher positions in the dominance hierarchies (LMM: Alpine/Forest Chi^2^ = 7.22, *p* = 0.007; Tree line/Forest Chi^2^ = 4.1, *p* = 0.043; Figure 6) than tree-line, or alpine grassland communities. Interestingly, CWM values of most (if not all) individual traits on the tree-line ecotone were closer to the trait distribution among the alpine grassland ant community rather than to the subalpine forest communities. This outcome is also in line with the results concerning species composition (see above).

## 4. Discussion

The recorded high ant species richness at the ecotone sites, compared to the adjacent alpine and forest sites, was expected. The putative reason in this particular case appeared to be the high incidence of several species of the Myrmicinae subfamily, instead of a “classical” mixing between two communities of bordering habitats. Only one single ant species (*Formica exsecta*) was found exclusively in alpine grassland and occurred just down to the tree line. This widespread grassland ant species is frequently observed in the Alps above the tree line, reaching about 2400 m elevation, but it is not a genuine specialist of high mountain environments [19,58,59]. All ant species occurring just inside the forest (four species at our sites) did not reach the tree line, although it is possible they did so in the vicinity of our sample locations. Typically, local ant assemblages at sites in the European Alps at comparable elevations comprise 2–9 species [5,6,10]. The numbers of ant species found on our sites were therefore in the expected range. Nevertheless, on a few sites in the alpine grassland zone (where up to eight species were detected to co-occur) local species richness was still quite high. Overall, gamma diversity was higher in the forest than in the alpine grassland zone, or directly at the tree line ecotone (Figure 3), indicating a larger potential species pool for forest sites than for the alpine grassland.

Community composition differed significantly among the habitats, with the community at the ecotone scoring more like the alpine grassland than the forest ant community. Elevation was, as expected, an important factor shaping the community composition over the ecotone. However, elevation is a proxy for general environmental change from the forest (at lower elevation) to the alpine sites (located at higher elevation), that comes along with a change in several life limiting factors for ants. Further, dwarf shrub cover scored as a significant factor, showing that habitat characteristics (such as high dwarf shrub cover directly at the tree line) significantly influenced the ant community composition especially at the ecotone.

Dwarf shrub cover and the humus indicator value derived from vegetation analysis significantly described the community of subordinate species, further underlining the importance of such local habitat characteristics on ant community composition. Further, wood ant abundance emerged as a significant factor influencing the community composition of the other subordinate ant species.

In alpine habitats, abiotic constraints appear to limit ant presence, mainly driven by the low ambient temperature [5,14]. However, for central-place foragers that live together in large societies like ants, competition is often considered as the major biotic force shaping communities [60]. A clear distinction between the relative roles of these factors along the gradient from subalpine forest to alpine grassland is not possible from a correlative field study, but the combination of results from our different analytical approaches may help disentangle their potential impact.

The overriding role of interspecific competition in shaping ant communities has recently been challenged [61], since it is hard to obtain unequivocal data about its real effects when no experimental data are available. A lack of co-occurrences noticed in multi-site field studies is not necessarily a sign of competition [61]. Nevertheless, several studies have concordantly found an inverse relation between the intensity of competition and elevation ([61] and citations therein). Hence, with a reduction in biotic constraints in less densely packed assemblages, abiotic filters are considered to act ever more severely with increasing elevation [5,13]. This shift in the relative importance of biotic to abiotic constraints was expected to shape the ant communities also over the rather short environmental gradient investigated here, from the subalpine forest to the alpine grassland. In line with this expectation, a high prevalence of mound-building wood ants was found in the subalpine forest (Figure 2) and their dominance indeed influenced the presence and abundance of the other ant species (Figure 4; Table 1), an already known effect of this dominant ant guild [33,62].

Yet, a recent study on ant communities in a temperate mixed hardwood forest in North America did not record a strong effect of a dominant *Formica* species on subordinate ant species [29]. Further, an interesting study in this same habitat [42] found resource availability to shape ant community composition (niche filtering effect) more strongly than competition among the species (niche partitioning). This conclusion, if applied to the present study, could indicate that the presence of honeydew-producing homopterans, bound to coniferous trees (and thus a higher availability of carbohydrates in the forest than in the alpine setting), might influence the community composition more severely (through a bottom-up control) than supposed competition [63]. In two previous studies on the same alpine habitat gradients as studied in the present paper, both a shift in the abundance of trophobiotic partners [31] and in the nutritional ecology of *Formica* ants [38] might also support the prime role of resource availability. Especially plant-derived resources, through the trophobiotic pathway, might be of central importance in shaping the ant community.

Overall, ant functional diversity scored highest at the ecotone sites indicating a greater functional breadth of the tree line ant community. The high species richness, known to correlate with functional richness [64], and the high abundance of the Myrmicinae species at the ecotone sites are expected to influence this pattern. Functional divergence was expected to peak in the forest, as a consequence of stronger character displacement (niche partitioning) among ant species co-occurring in the forest, thereby mitigating direct competition among species [65]. This expectation was however not met in the present study, adding to the doubts that competition by wood ant is indeed the most decisive driving force in the here investigated community composition.

Considerations of the CWMs of species traits related to the feeding preferences among the ant communities revealed a reduction in carnivorous feeding habits and an increase in trophobiosis, when descending from the alpine grassland to the forest. Hence, despite the somewhat coarse quality of available data (ant abundance scores and qualitative literature records on feeding preferences [18]), a clear ecological pattern emerged. The high score of carnivory on the alpine and tree line sites is clearly complementary to trophobiotic feeding habits. All the presented traits fit well to the notion of an increased competition pressure in the forest and are clearly related to the increasing abundance of mound-building wood ants in the forest. Hence, prevalence of wood ants is related to a shift in the abundances of traits and accordingly to a change in the overall ecological role of ants over the short environmental gradient along the alpine tree-line. Nevertheless, the shift in feeding habits might also mirror the community-wide response among ants to a resource gradient, e.g., linked to the availability of trophobionts (as recorded by [42]).

Climate change will probably shift the tree line ecotone upwards [22] and ant species distribution will probably change, too. New species from lower elevation might join the tree line ant community before it shifts its location upwards, tracking the up-slope shift of trees. The competition pressure within the ant community might then change, for example if *Lasius (Dendrolasius) fuliginosus* (Latreille, 1798) as a dominant species starts competing with wood ants also at higher elevations. However, the more prominent and unforeseeable shift might happen in the complex trophic relation between ants, trophobionts and plants that appear to be essential in shaping the tree line ant community.

While our correlative approach is not the optimal way to evaluate if competition or resource availability is the prime force shaping the community composition and in our study competitive interactions were not targeted directly, our results leave good reason to infer that both aspects play an important role. Further investigations that address specific community assembly rules, analyzing competitive interactions, or quantifying resource availability and use, could shed more light on this very interesting and poorly studied topic: What really drives ant community composition over an elevation gradient? Is competition (niche partitioning) or rather resource availability (niche filtering) the more important process? This debate is still not settled.

## 5. Conclusions

The ecotone sites situated directly at the alpine tree-line harbored a high species richness, but this resulted from a denser species packing rather than from faunal mixing at the intersection of two distinct habitat types. Ant community composition was significantly influenced by elevation and habitat attributes, such as dwarf shrub cover and humus. Further, the community of subordinate ants was influenced by the abundance of mound-building dominant red wood ants. The effect of wood ants in shaping both the ant community composition and its functional dimension appeared as an important biotic constraint (niche partitioning effect), although we did not test this directly. Niche filtering through the availability of trophobionts bound to coniferous trees appears to represent a plausible alternative explanation for the observed biodiversity patterns.

## Figures and Tables

**Table 1:** Model selection results for distance-based redundancy analysis (*capscale()* function in R package vegan) of ant assemblage composition, first for the full ant community, and additionally for the community of subordinate ant species. Three putative explanatory variables were tested with addition of the influence of wood ant abundance on the subordinate community. Mountain slope identity was always modelled as random factor to control for spatial autocorrelation. AICc defines the Akaike information criterion corrected for small sample sizes, *w* the Akaike weights and R^2^ the adjusted explained variance. The best model is printed in bold face.

Fixed Factors	Nr. Variables	Full Community			Community without Wood Ants		
		AICc	*w*	R^2^ adj.	AICc	*w*	R^2^ adj.
Wood ants + Humus + Shrub + Elevation	5				30.12	0.08	0.41
Wood ants + Humus + Shrub	4				**28.49**	**0.** **19**	**0.** **40**
Wood ants + Humus + Elevation	4				31.98	0.03	0.28
Wood ant + Shrub + Elevation	4				30.77	0.06	0.32
Humus + Shrub + Elevation	4	30.54	0.16	0.32	29.82	0.10	0.36
Humus + Shrub	3	31.69	0.09	0.22	29.87	0.09	0.30
Humus + Elevation	3	31.38	0.11	0.23	31.61	0.04	0.23
Shrub + Elevation	3	**29.17**	**0.32**	**0.31**	30.54	0.07	0.27
Wood ants + Elevation	3				32.14	0.03	0.21
Wood ants + Humus	2				30.53	0.07	0.27
Wood ants + Shrub	2				29.75	0.10	0.30
Wood ants	2				31.61	0.04	0.18
Humus	2	32.30	0.07	0.14	31.48	0.04	0.18
Shrub	2	32.70	0.06	0.13	32.86	0.02	0.13
Elevation	2	30.41	0.17	0.21	31.82	0.04	0.17
Null Model	1	34.53	0.02	0	34.45	0.01	0

## Data Availability

The data presented in this study are available in Supplementary Material.

## Supplementary Materials

The following are available online at https://www.mdpi.com/2075-4450/12/3/219/s1, Table S1: Site information, Table S2: Ant species list, Table S3: Trait list.
