# Supplementary material for: Ant Diversity and Community Composition in Alpine Tree Line Ecotones
Source: Insects. 2021 Mar 4;12(3):219. doi: 10.3390/insects12030219 (PMC8001821; doi:10.3390/insects12030219)
Supplement: Supplementary file 1 [file insects-12-00219-s001.zip › Table S2.docx]

**Ant species list**

Table S2. Ant species presence on the five mountains (indicated by the letters M, O, T, U & M) and three habitats (A: Alpine grassland; T: Tree-line; F: forest). Numbers refer to the pseudo-abundance score of ant species on each site (ranging from 0-5).

| **Species** | **Slope** | **M** | **M** | **M** | **M** | **M** | **O** | **O** | **O** | **O** | **O** | **T** | **T** | **T** | **T** | **T** | **U** | **U** | **U** | **U** | **U** | **Z** | **Z** | **Z** | **Z** | **Z** |
| --- | --- | --- | --- | --- | --- | --- | --- | --- | --- | --- | --- | --- | --- | --- | --- | --- | --- | --- | --- | --- | --- | --- | --- | --- | --- | --- |
|  | **Habitat** | **A** | **A** | **T** | **F** | **F** | **A** | **A** | **T** | **F** | **F** | **A** | **A** | **T** | **F** | **F** | **A** | **A** | **T** | **F** | **F** | **A** | **A** | **T** | **F** | **F** |
| *Camponotus herculeanus* (Linnaeus, 1758) | | 0 | 0 | 0 | 0 | 0 | 0 | 0 | 0 | 0 | 1 | 0 | 0 | 0 | 0 | 0 | 0 | 0 | 0 | 0 | 0 | 0 | 0 | 0 | 0 | 0 |
| *Formica aquilonia* Yarrow, 1955 (wood ant) | | 0 | 0 | 0 | 0 | 0 | 0 | 0 | 0 | 5 | 3 | 0 | 0 | 0 | 0 | 5 | 0 | 0 | 0 | 0 | 2 | 0 | 0 | 0 | 0 | 0 |
| *Formica exsecta* Nylander, 1846 | | 0 | 2 | 0 | 0 | 0 | 0 | 0 | 0 | 0 | 0 | 5 | 5 | 2 | 0 | 0 | 0 | 1 | 1 | 0 | 0 | 2 | 0 | 1 | 0 | 0 |
| *Formica lemani* Bondroit, 1917 (slave ant) | | 5 | 5 | 5 | 5 | 4 | 5 | 5 | 5 | 1 | 4 | 5 | 5 | 5 | 4 | 0 | 5 | 5 | 5 | 4 | 1 | 5 | 5 | 5 | 4 | 5 |
| *Formica lugubris* Zetterstedt, 1838 (wood ant) | | 4 | 0 | 4 | 4 | 3 | 4 | 3 | 3 | 0 | 1 | 0 | 0 | 1 | 5 | 0 | 4 | 4 | 4 | 4 | 5 | 0 | 4 | 5 | 5 | 4 |
| *Leptothorax acervorum* (Fabricius, 1793) | | 1 | 0 | 1 | 1 | 1 | 0 | 0 | 1 | 0 | 1 | 0 | 1 | 1 | 0 | 0 | 0 | 0 | 3 | 2 | 0 | 2 | 2 | 0 | 1 | 2 |
| *Manica rubida* (Latreille, 1802) | | 4 | 2 | 5 | 0 | 4 | 0 | 1 | 2 | 0 | 1 | 0 | 0 | 2 | 0 | 1 | 1 | 2 | 5 | 1 | 0 | 0 | 2 | 3 | 2 | 2 |
| *Myrmica lobicornis* Nylander, 1846 | | 0 | 0 | 0 | 0 | 1 | 0 | 0 | 0 | 0 | 0 | 0 | 0 | 0 | 0 | 0 | 0 | 0 | 0 | 0 | 0 | 0 | 0 | 0 | 0 | 0 |
| *Myrmica lobulicornis* Nylander, 1857 | | 1 | 1 | 2 | 4 | 2 | 2 | 1 | 2 | 1 | 0 | 1 | 1 | 1 | 1 | 0 | 5 | 3 | 3 | 2 | 1 | 1 | 1 | 2 | 0 | 3 |
| *Myrmica ruginodis* Nylander, 1846 | | 0 | 0 | 0 | 0 | 0 | 0 | 0 | 0 | 0 | 0 | 0 | 0 | 0 | 0 | 0 | 0 | 0 | 0 | 0 | 0 | 0 | 0 | 0 | 1 | 2 |
| *Myrmica sulcinodis* Nylander, 1846 | | 2 | 0 | 0 | 1 | 1 | 0 | 0 | 2 | 1 | 1 | 0 | 0 | 0 | 1 | 1 | 1 | 1 | 1 | 1 | 0 | 0 | 4 | 1 | 0 | 2 |
| *Tetramorium alpestre* Steiner et al., 2010 | | 4 | 3 | 2 | 1 | 0 | 3 | 3 | 2 | 0 | 1 | 0 | 0 | 2 | 0 | 0 | 0 | 1 | 1 | 0 | 1 | 1 | 1 | 2 | 0 | 3 |
| *Temnothorax tuberum* Fabricius, (1775) | | 0 | 0 | 2 | 1 | 0 | 2 | 2 | 2 | 0 | 0 | 0 | 0 | 0 | 0 | 0 | 0 | 1 | 2 | 0 | 0 | 0 | 2 | 2 | 1 | 3 |
