# Supplementary material for: Ant Diversity and Community Composition in Alpine Tree Line Ecotones
Source: Insects. 2021 Mar 4;12(3):219. doi: 10.3390/insects12030219 (PMC8001821; doi:10.3390/insects12030219)
Supplement: Supplementary file 1 [file insects-12-00219-s001.zip › Table S1.doc]

**Site information**

Table S1. Site information and variables implemented in the constrained ordination analysis of ant community composition (excluding mound-building wood ants).

| Plot code | Slope | Habitat | Longitude | Latitude | Exposition | Elevation (m a.s.l.) | Tree cover | Draw shrub cover | Ground vegetation cover | Bare soil cover | Rocks cove | Dead wood cover | Average humus indicator value of vascular plants | Average light indicator value of vascular plants |
| --- | --- | --- | --- | --- | --- | --- | --- | --- | --- | --- | --- | --- | --- | --- |
| Z 1 | Zielspitz | alpine | N46°41.359' | E011°01.829' | SSE | 2239 | 0 | 0.1 | 0.8 | 0.02 | 0.1 | 0 | 3 | 4 |
| Z 2 | Zielspitz | alpine | N46°41.331' | E011°01.942' | SE | 2186 | 0 | 0.075 | 0.75 | 0.05 | 0.125 | 0 | 3 | 4 |
| Z 3 | Zielspitz | tree line | N46°41.283' | E011°01.968' | S | 2130 | 0.275 | 0.15 | 0.625 | 0.025 | 0.1 | 0.01 | 3 | 4 |
| Z 4 | Zielspitz | forest | N46°41.241' | E011°01.941' | SSW | 2082 | 0.625 | 0.2 | 0.45 | 0.155 | 0.03 | 0.03 | 3 | 3 |
| Z 5 | Zielspitz | forest | N46°41.179' | E011°01.926' | SSW | 2015 | 0.45 | 0.225 | 0.35 | 0.1 | 0.1 | 0.03 | 3 | 3 |
| O 1 | Orgelspitz | alpine | N46°33.218' | E010°44.864' | SE | 2393 | 0 | 0.425 | 0.45 | 0.015 | 0.11 | 0 | 3 | 4 |
| O 2 | Orgelspitz | alpine | N46°33.201' | E010°44.944' | SE | 2339 | 0 | 0.55 | 0.35 | 0.035 | 0.075 | 0 | 3 | 4 |
| O 3 | Orgelspitz | tree line | N46°33.149' | E010°45.060 | SSE | 2270 | 0.3 | 0.55 | 0.3 | 0.04 | 0.06 | 0.025 | 3 | 4 |
| O 4 | Orgelspitz | forest | N46°33.033' | E010°45.013' | SSE | 2210 | 0.6 | 0.125 | 0.525 | 0.11 | 0.15 | 0.06 | 4 | 3.5 |
| O 5 | Orgelspitz | forest | N46°32.926 | E010°44.839' | SSE | 2166 | 0.5 | 0.2 | 0.35 | 0.2 | 0.175 | 0.05 | 5 | 3 |
| U 1 | Ulten | alpine | N46°30.630' | E010°51.043' | S | 2352 | 0 | 0.45 | 0.425 | 0.04 | 0.11 | 0.005 | 3 | 4 |
| U 2 | Ulten | alpine | N46°30.519' | E010°51.103' | S | 2280 | 0 | 0.425 | 0.5 | 0.045 | 0.055 | 0.005 | 3 | 4 |
| U 3 | Ulten | tree line | N46°30.440' | E010°51.168' | SSE | 2230 | 0.25 | 0.35 | 0.4 | 0.075 | 0.06 | 0.02 | 3 | 4 |
| U 4 | Ulten | forest | N46°30.374' | E010°51.235' | SE | 2186 | 0.65 | 0.3 | 0.55 | 0.085 | 0.03 | 0.06 | 5 | 3 |
| U 5 | Ulten | forest | N46°30.279' | E010°51.354' | SSE | 2111 | 0.625 | 0.1 | 0.35 | 0.35 | 0.05 | 0.075 | 3 | 3 |
| T 1 | Tschigat | alpine | N46°42.757' | E011°03.916' | S | 2231 | 0 | 0 | 0.95 | 0 | 0.05 | 0 | 3 | 4 |
| T 2 | Tschigat | alpine | N46°42.719' | E011°03.900' | S | 2180 | 0 | 0.125 | 0.625 | 0 | 0.25 | 0 | 3 | 4 |
| T 3 | Tschigat | tree line | N46°42.598' | E011°03.833' | SSW | 2090 | 0.375 | 0.4 | 0.45 | 0.1 | 0.1 | 0.01 | 3 | 3 |
| T 4 | Tschigat | forest | N46°42.521' | E011°03.802' | SSW | 1987 | 0.625 | 0.125 | 0.45 | 0.035 | 0.15 | 0.015 | 5 | 3 |
| T 5 | Tschigat | forest | N46°42.433' | E011°03.851' | S | 1935 | 0.7 | 0.1 | 0.55 | 0.2 | 0.1 | 0.05 | 5 | 3 |
| M 1 | Martell | alpine | N46°30.662' | E010°42.589' | SSE | 2405 | 0 | 0.5 | 0.4 | 0.05 | 0.075 | 0.005 | 3 | 4 |
| M 2 | Martell | alpine | N46°30.588' | E010°42.673' | SSE | 2349 | 0 | 0.4 | 0.525 | 0.035 | 0.075 | 0 | 3 | 4 |
| M 3 | Martell | tree line | N46°30.495' | E010°42.700' | SSE | 2300 | 0.4 | 0.45 | 0.425 | 0.065 | 0.03 | 0.04 | 3 | 4 |
| M 4 | Martell | forest | N46°30.461' | E010°42.789' | SSE | 2260 | 0.55 | 0.15 | 0.5 | 0.15 | 0.125 | 0.1 | 3 | 3 |
| M 5 | Martell | forest | N46°30.423' | E010°42.820' | SSE | 2205 | 0.6 | 0.05 | 0.45 | 0.175 | 0.1 | 0.125 | 5 | 4 |
